# H_2_ clumped isotope measurements at natural isotopic abundances

**DOI:** 10.1002/rcm.8323

**Published:** 2019-01-11

**Authors:** Maria Elena Popa, Dipayan Paul, Christof Janssen, Thomas Röckmann

**Affiliations:** ^1^ Institute for Marine and Atmospheric Research Utrecht (IMAU) Utrecht University Princetonplein 5 Utrecht 3584CC The Netherlands; ^2^ Centre for Isotope Research (CIO) University of Groningen Nijenborgh 6 Groningen 9747AG The Netherlands; ^3^ Observatoire de Paris, Université PSL, CNRS, LERMA‐IPSL, Laboratoire d'Etudes du Rayonnement et de la Matière en Astrophysique et Atmosphères Sorbonne Université 77 Avenue Denfert‐Rochereau Paris 75014 France

## Abstract

**Rationale:**

Molecular hydrogen (H_2_) is an important gas for atmospheric chemistry, and an indirect greenhouse gas due to its reaction with OH. The isotopic composition of H_2_ (δD) has been used to investigate its atmospheric budget; here we add a new observable, the clumped isotopic signature ΔDD, to the tools that can be used to study the global cycle of H_2_.

**Methods:**

A method for determining ΔDD in H_2_ was developed using the high‐resolution MAT 253‐Ultra isotope ratio mass spectrometer (Thermo Fisher). The HH, HD and DD abundances are quantified at medium resolution (M/ΔM ≈ 6000), which is sufficient for HD^+^ and DD^+^ to be distinguished from H_3_^+^ and H_2_D^+^, respectively. The method involves sequential measurement of isotopologues, and DD is measured using an ion counter. For verification, catalytic ΔDD equilibration experiments were performed at temperatures of up to 850°C.

**Results:**

The typical precision obtained for ΔDD is 2–6‰, close to the theoretical counting statistics limit, and adequate for detecting the expected natural variations. Compatibility and medium‐term reproducibility are consistent with the precision values. The method was validated using temperature equilibration experiments, which showed a dependence of ΔDD on temperature as expected form theoretical calculations.

**Conclusions:**

We have established a method for determining ΔDD in H_2_ at natural isotopic abundances, with a precision that is adequate for observing the expected variations in atmospheric and other natural H_2_. This method opens the road to new research on the natural H_2_ cycle.

## 1 BACKGROUND AND INTRODUCTION

Molecular hydrogen (H_2_) is an important component of the terrestrial system, present in the atmospheric, oceanic, geologic, and biologic components. In the atmosphere, at a mole fraction of about 500 ppb, H_2_ is the second most abundant reduced gas after methane, and it has a lifetime of about 2 years. H_2_ is produced by incomplete combustion processes, together with CO, and from atmospheric oxidation of CH_4_ and non‐methane hydrocarbons via the intermediate formaldehyde.^1^, ^2^ It reacts with atmospheric OH radicals, reducing the OH abundance, thus increasing the lifetime of CH_4_. Because of that, H_2_ is considered an indirect greenhouse gas.^3^

Atmospheric H_2_ is studied because of its relevance for atmospheric composition and chemistry. However, H_2_ can also be a tool for understanding other aspects of nature –e.g., the temperature inside volcanoes, microbial metabolism, or human health. Being the simplest molecule, it is also used for fundamental chemistry and physics studies.^4^, ^5^

H_2_ has a strong link with microbial life; for example, it is produced in soils and in the ocean in the process of bacterial N_2_ fixation, and in microbial fermentation processes. It is an important subsistence source of energy for soil microbes, which makes soil uptake the most important sink for atmospheric H_2_;^2^, ^6^, ^7^ it is also an important source of energy for other (chemoautotrophs, extremophiles) microbes.^8^, ^9^, ^10^ It is involved in anoxic methane formation, for example in wetlands. H_2_ is also produced by bacteria in the digestive tract of living animals, including humans, and anomalous H_2_ production is used in medicine for diagnosis.^11^, ^12^ Furthermore, H_2_ can be produced by geologic processes. Studying the abundance and isotopic composition of H_2_ emitted from seeps and volcanoes can provide information about underground processes and temperatures.^13^, ^14^, ^15^, ^16^ In addition to its natural relevance, H_2_ is also important economically as a clean energy carrier, and its use is expected to increase in the future.^3^, ^17^, ^18^

Hydrogen has two stable isotopes, protium (^1^H) and deuterium (^2^H or D). The protium nucleus has only one proton and no neutrons (atomic mass 1) and it accounts for about 99.984% of all hydrogen atoms in water on Earth. Deuterium has one proton and one neutron (atomic mass 2) and its natural relative abundance on Earth is 0.0156%. The molecules of H_2_ can consist of any combination of these two atoms, i.e. HH, HD or DD; the DD molecules are the so‐called “clumped isotope” variants. If the atoms combine in a purely stochastic way, the abundance of HD molecules will be 0.03%, i.e. double the abundance of D atoms (because both HD and DH molecules are formed, each with a probability of 0.015%). The relative abundance of the DD molecules will be 2.4E‐8, calculated as (D/H)^2^.

The different H_2_ isotopologues have different masses and, because of this, slightly different physical and chemical behavior; this leads to discrimination during physical or chemical processes and thus to isotopic fractionation. Based on this, analyzing the isotopic composition of H_2_ can help understand various aspects of the natural H_2_ cycle. Recently, it became possible to analyze the D content of H_2_ (δD) in small atmospheric samples at natural isotopic abundances.^19^, ^20^ These measurements enabled the study of different components of the H_2_ cycle^21^, ^22^, ^23^, ^24^ and helped constrain the atmospheric H_2_ budget, which as a consequence is currently relatively well understood on the large scale.^6^, ^25^, ^26^ Although H_2_ isotope measurements have proven useful, they are difficult to perform, and only few labs had or have this capability.

The δD value quantifies the abundance of D atoms in the sample and it is estimated from the measurements of HD/H_2_ molecular ratio. DD molecules are also present in natural H_2_, but at such a low abundance that they do not contribute significantly to the total D quantity. However, the DD content in H_2_ is a potentially interesting and independent tracer. At stochastic distribution of isotopes among molecules, the DD content is related to the HD content, and thus to the δD value. However, at ambient temperatures formation of DD molecules is thermodynamically favored over HD, because the zero‐point energy is lower for the left‐hand side of the exchange reaction below:

HH+DD↔2HD

At thermodynamic equilibrium, DD will be enriched compared with the amount corresponding to a stochastic distribution. Thus, while the total D proportion stays constant, at different temperatures the D atoms are distributed between HD and DD in different fractions. At high temperatures (>1000°C) the thermodynamic equilibrium tends towards the stochastic distribution HD^2^/HHxDD = 4. It is interesting to note that the equilibrium has been used as a prime example for illustrating the principle of clumped isotope fractionation in the past,^27^ although multiply substituted isotopologues have never been measured at natural abundance before, only when enriched mixtures were used.^28^, ^29^, ^30^

The distribution of the D atoms between the HD and DD molecules is quantified by the ΔDD value (see section 2 for definition). For H_2_ in thermodynamic equilibrium, according to classical isotope theory, ΔDD is expected to be strongly temperature dependent, with a temperature coefficient of about −1‰ per °C at ambient temperatures^5^ (see also Figure 6). Thus, when the sample can be assumed to be in thermodynamic equilibrium, ΔDD can be used to derive the temperature of equilibration (or formation). Such situations occur, for example, in geological or combustion processes. It is also possible that H_2_ is not in thermodynamic equilibrium; in such situations, if the formation temperature is known, the departure from equilibrium may provide information on the underlying processes occurring during H_2_ formation. Examples of potential applications are (micro)biological and chemical (kinetic) processes, when the temperatures are known.

The expected ΔDD variations in natural H_2_ are of tens or hundreds of ‰, due to the large mass and energy difference of the isotopologues and to the large variation in ΔDD with temperature.^5^ Large ΔDD signals can also occur from mixing H_2_ gases with different isotopic compositions, even if, for each of these gases, the ΔDD = 0.^31^ Finally, when forming H_2_ from two atom pools with different isotope ratios, a negative ΔDD value can result.^32^, ^33^

The clumped isotope ratio of molecular hydrogen at natural abundances has never been measured. That is partly because of the very low abundance of the DD molecules. Furthermore, the DD molecule and the ^4^He molecule interfere at the same nominal mass 4 and are not distinguishable using conventional isotope ratio mass spectrometry (IRMS) instruments. However, instruments with much higher mass resolution have become available in recent years. This has opened up the possibility to measure new isotopic signatures, including the very rare clumped isotopes.

In this paper we report the first high‐precision (‰ level) measurements of the abundance of DD in H_2_ at natural isotopic composition, using the new high‐resolution, high‐sensitivity MAT 253‐Ultra IRMS. We describe the measurement method, which is based on rapid sequential measurement of HH, HD and DD, and evaluate the performance. We also present isotope equilibration experiments at different temperatures that we performed for validation, and compare them with the temperature dependence expected from theoretical calculations.

## 2 METHODS

### 2.1 Definitions and units

D/H and DD/HH ratios are reported using the δ values, defined as follows:

(1) $$\mathrm{\delta D}=\frac{{\left(D/H\right)}_{\text{sample}}}{{\left(D/H\right)}_{\text{reference}}}-1$$

(2) $$\mathrm{\delta DD}=\frac{{\left(\mathrm{DD}/\mathrm{HH}\right)}_{\text{sample}}}{{\left(\mathrm{DD}/\mathrm{HH}\right)}_{\text{reference}}}-1$$

The international reference is Vienna Standard Mean Ocean Water (VSMOW), with a D/H ratio of (155.76 ± 0.8) × 10^−6^.

The clumped isotope value ΔDD is calculated as the relative difference between the actual DD/HH ratio and the stochastic DD/HH ratio; the stochastic ratio is calculated from the D/H content, assuming stochastic distribution of H and D atoms in HH, HD and DD molecules.

(3) $$\mathrm{∆DD}=\frac{\left(\mathrm{DD}/\mathrm{HH}\right)}{{\left(\mathrm{DD}/\mathrm{HH}\right)}_{\text{stochastic}}}-1=\frac{\left(\mathrm{DD}/\mathrm{HH}\right)}{{\left(D/H\right)}^{2}}-1$$

The δD and δDD values represent the relative abundances of D atoms and DD molecules, respectively. ΔDD refers instead to the distribution of the D atoms among HD and DD molecules, and it is independent of the abundance of D in the sample. Note that the stochastic HD/HH ratio is twice the D/H ratio, because there are two different sites where a D can replace an H in the H₂ molecule, each with a D/H chance of occurrence. Therefore, when relating the abundances of DD and HD molecules,

(4) $$\mathrm{∆DD}=\frac{\left(\mathrm{DD}/\mathrm{HH}\right)}{{\left(D/H\right)}^{2}}-1=4\times \frac{\left(\mathrm{DD}/\mathrm{HH}\right)}{{\left(\mathrm{HD}/\mathrm{HH}\right)}^{2}}-1$$

### 2.2 Instrument

The high‐resolution isotope ratio mass spectrometer MAT253‐Ultra (Thermo Fisher Scientific GmbH, Bremen, Germany) at IMAU (Institute for Marine and Atmospheric Research Utrecht, The Netherlands) has nine detector units (L4‐L1, Center, H1‐H4), all equipped with Faraday cups. Three additional miniature ion counters (compact discrete dynodes, CDDs) are placed adjacent to the last three Faraday cups on the high‐mass side of the detector array (H2, H3 and H4), and the center position is additionally equipped with a retarding potential quadrupole secondary electron multiplier (RPQ‐SEM). Except for the detectors in the center position, all the detectors are movable, thus allowing the instrument to be configured for measuring different species. In addition, the Faraday cups can be configured with any of the available amplifiers (3 × 10^8^ Ω, 1 × 10^9^ Ω, 1 × 10^10^ Ω, 1 × 10^11^ Ω, 1 × 10^12^ Ω and 1 × 10^13^ Ω), depending on the intensity of the measured ion beam. The instrument can be used in low, medium or high mass resolution; the resolution is controlled by the size of the entrance slit used (250, 16, and 5 μm for low, medium and high resolution, respectively), and higher mass resolution is associated with lower signal intensity. For this work we used the medium resolution setting, which gives for H_2_ a mass resolving power of around 6000. Here we use the M/ΔM definition of mass resolving power, where ΔM corresponds to the mass width of the rising/falling edge of a peak, between 5% and 95% of the maximum peak intensity.^34^ There are two dual‐inlet systems, each with two adjustable‐volume (max. 40 mL) metal bellows.

Qtegra (Thermo Fisher Scientific) is the software that controls the instrument and makes a preliminary data analysis; during the time of this work it was still under development. The prototype of the MAT 253‐Ultra has previously been described by Eiler et al.^34^

### 2.3 Isotopologues and peak shapes

The isotopologues of interest are HH, HD and DD, and their average natural abundances are shown in Table 1. Also shown in this table are the typical number of counts per second (cps) for our main H_2_ reference gases at normal working source pressure of 2.5 × 10^−7^ mbar, and the corresponding counting statistics precision. The IMAU‐H_2_ reference gas has a δD value of about −150‰, and DD equilibrated at room temperature. The counting statistics precision limit (shot noise) is calculated as the inverse square root of the total number of counts over the integration time considered. Because of the low abundance of DD, we needed to maximize the signal, and thus to use the maximum possible source pressure. The working source pressure of 2.5 × 10^−7^ mbar was chosen to keep the balance between maximizing the DD counts and maintaining a reasonable filament lifetime.

**Table 1:** Average natural abundance and molecular masses of the three molecules of interest; intensity in cps for the IMAU‐H_2_ reference, and the corresponding counting statistics precision limit for the given integration times

Isotopologue	Natural abundance (%)*	Molecular mass	Intensity in cps (IMAU‐H_2_)	Integration time (s)	Counting statistics std (‰)
HH	99.9688	2.0157	9 × 10^9^	1	0.01
HD	0.0312	3.0219	3 × 10^6^	10	0.18
DD	2.43 × 10^−6^	4.0282	100	1000	3.16

* for DD, this is the abundance at stochastic distribution.

**Figure 1:**
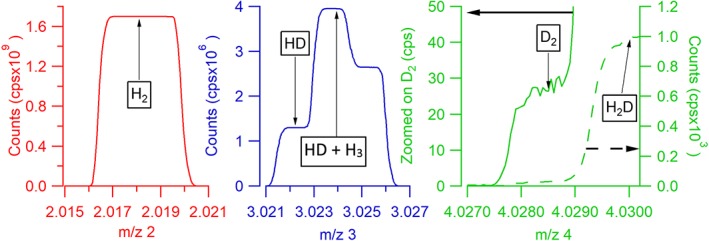
Mass scans around the HH, HD and DD peaks. The HD and DD peaks partly overlap with the adduct peaks H_3_ and H_2_D; the plateaus where HD and DD can be measured have a width of about 1 mu only [Color figure can be viewed at wileyonlinelibrary.com]

Medium‐resolution scans of the peaks at the corresponding masses are shown in Figure 1. The HD and DD peaks partially overlap with H_3_ and H_2_D, respectively, which are both adducts formed inside the source. The plateaus where the individual HD and DD species are measured (see arrows in Figure 1) are only about 1 milli unified mass unit (mu) wide. The signal of the DD isotopologue shown in Figure 1 is less than 30 cps – these scans were made using a different gas from the one shown in Table 1, and a different source pressure.

### 2.4 Measurement method

The measurement of the three molecules of interest (HH, HD, DD) cannot be performed at the same time, because the large relative difference between the three isotopologue masses results in a dispersion of the three ion beams that is larger than the collector plane of the instrument (dispersion of ~15%). Thus for H_2_ we developed a method in which the different isotopologues are measured sequentially.

In this measurement method, we define a separate collector configuration for each isotopologue, and the measurement sequence cycles through these three configurations. Each step involves adjusting the magnetic field ‐ this will be referred to as “peak hopping” in the following. Although this method in principle requires only one detector, the wide dynamic range of ion intensities for the different isotopologues requires use of an ion counter for DD and of Faraday collectors for HH and HD. The present method uses three different collectors (L3 with a 10^9^ Ω amplifier, H1 with a 10^12^ Ω amplifier and H3 with a CDD ion counter) in order to minimize the necessary change of the magnetic field and to avoid exchanging the amplifiers.

**Figure 2:**
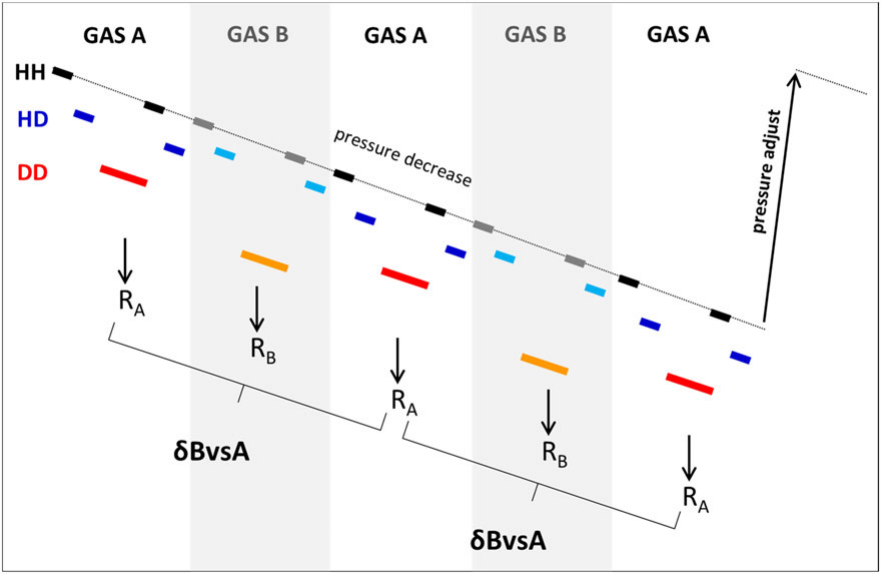
Conceptual illustration of the measurement sequence (figure not to scale). One measurement cycle contains a series A‐B‐A‐B‐A, with A and B being the two gases analyzed. Each of the gases A and B is analyzed for the three masses successively, with different integration times needed for each mass to attain the necessary precision. As the pressure is decreasing, the three isotopologues are analyzed at different pressures. The HD/HH and DD/HH ratios are calculated for each gas, and then the δ value of the sample (gas B in this case) is calculated relative to the bracketing reference measurements [Color figure can be viewed at wileyonlinelibrary.com]

The isotope ratios are determined for a sample versus a reference gas. The measurement sequence is shown in Figure 2. One measurement cycle contains a series A‐B‐A‐B‐A, with A and B being the two gases analyzed. Before each cycle, the gas pressures in the bellows of the dual‐inlet system are adjusted to reach the desired signal intensity. Each of the gases A and B is analyzed for the 3 masses successively, with different integration times needed for each mass to attain the necessary precision. The measurement of DD requires a short “dummy” measurement series for the CDD stabilization. This is followed by 5 long (33 s) analyses, which are averaged, resulting in one data point. The HD/HH ratio is determined for each gas before and after the DD measurement, as a check for memory effects when switching between the sample and reference gas.

The final δ values are calculated from the isotope ratios of the sample and the bracketing reference gas.

This method poses particular challenges to the stability and performance of the instrument, which are conceptually different from conventional isotope ratio measurements that use multiple collector arrays where the different isotopologues are recorded simultaneously.

- After measurement of each individual isotopologue, the magnetic field has to be changed in order to record the following isotopologue. The mass jumps have to be reproducible at the ~0.1 mu level for HD and DD because these isotopologues are measured on a narrow peak shoulder (Figure 1). In the MAT 253‐Ultra instrument this is facilitated using a high‐precision magnetic field probe.
- Both the source and the bellow pressure decrease continuously, because the gas is being consumed. Since the isotopologues are measured successively and not simultaneously as in conventional IRMS measurements, the pressure changes from one isotopologue measurement to the next. The isotope ratios and the δ values have to be determined from these dynamically changing signals. This is taken into account by referencing the ratios of gas B to the average of the surrounding ratios of gas A, which mostly cancels the systematic error in ratios due to the pressure decrease.

As the flat areas of the pure HD and DD peaks are very narrow (about 1 mu), the stability of the peak position in time is very important. A scan for each mass is performed before starting any new analysis, after which the mass positions are adjusted in the cup configuration. During longer measurements, the stability of the mass position is usually verified by including a “narrow mass‐range sweep” in each cycle, which is a scan over the mass range surrounding the rising edge of the peak of interest. In our case, the change in the peak position is usually similar for the three masses; thus a sweep around mass 3 is sufficient for assessing the stability. Mass scale instabilities are still one of the limiting factors for successful analyses.

The equilibration time when switching between two gases (from two bellows) is set at 10 s. The time after which the H_2_ in the source is removed was tested by filling the source with H_2_ sample at the normal working pressure, and then closing the sample inlet valve to the source and monitoring the removal of the gas from the source. The signal intensity dropped to less than 0.05% of the initial count within 2 s with the S‐window closed (less than 0.02 in 2 s with the S‐window open). For the H_2_ measurements, the S‐window is closed.

### 2.5 Gases

For setting up and testing the measurement method, we used a number of H_2_ gases and mixtures with different isotopic composition. The δD scale is maintained using two small calibration H_2_ cylinders (Messer Griesheim, Bad Soden, Germany), with nominal δD values of −200 and +200‰ vs VSMOW. Our lab reference (IMAU‐H_2_) cylinder has a δD value of about −150‰. No gases with known δDD (and ΔDD) values were available. Our measurements showed that two of the cylinders (the laboratory reference gas and the small cylinder with δD value of −200‰) had a DD composition suggesting equilibration at ambient temperature, while the small cylinder with δD value of +200‰ (NAT‐360) was extremely enriched in DD, with a ΔDD anomaly of about +26000‰. This is probably because the positive δD value was reached by spiking more D‐depleted H_2_ with isotopically labeled HD that also contained a significant amount of DD. Using these gases, we also produced a series of mixtures with different isotopic compositions.

For mixing and storing the H_2_ gases, we used several types of glass bottles, including the 1‐L glass flasks (Normag, Limenau, Germany) that we normally use for atmospheric samples. All these containers have valves with Teflon or PCTFE (polychlorotrifluoroethylene) tips, without O‐rings, and during our tests over several months they did not alter the isotopic composition of the H_2_ stored.

A summary of the gases used is given in Supplement 1 (supporting information).

### 2.6 Experiments

In order to optimize and test the H_2_ measurement method, about 150 experiments were performed, mostly between July and September 2017. Most experiments involved analyzing two gases (different or not), from two bellows, against each other.

The experiments were carried out to establish:

- instrumental precision under different conditions (e.g. at different source pressures).
- medium‐term reproducibility for the same sample (weeks to months).
- consistency of results between different samples.
- temperature equilibration (see section 2.6.1).

An overview of all experiments is given in Supplement 2 (supporting information).

#### 2.6.1 Heating experiments

According to theory, at thermodynamic equilibrium, ΔDD is strongly dependent on temperature. We performed a series of heating experiments, in which we equilibrated H_2_ samples at various temperatures, and compared the results with the theoretical temperature dependence calculated as described in section 2.7. The main purpose of these experiments was to validate our ΔDD measurement method, but also to preliminarily test a method that we intend to employ in the future for producing calibration gases with known ΔDD. A similar thermodynamic equilibrium gas‐based calibration approach has been applied for other clumped isotope measurements, e.g. CO_2_, CH_4_, O_2_, N_2_.^35^, ^36^, ^37^, ^38^, ^39^

**Figure 3:**
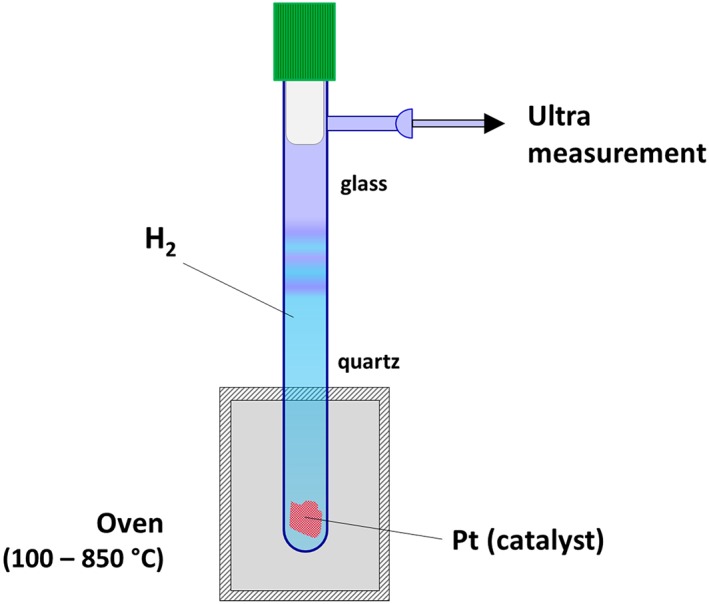
Schematic of the setup used for the heating experiments. The heating tube is made partly of quartz (the heated part) and partly of glass (the upper part). The tube is connected to the IRMS setup and the H_2_ is expanded directly to the bellows for measurement after the desired heating time [Color figure can be viewed at wileyonlinelibrary.com]

For these experiments we used two “heating tubes” which have the heated part made of quartz and the cold part (including the valve and connection) made of borosilicate glass (Figure 3). The tube has a small piece of Pt mesh at the bottom, which functions as a catalyst for the H_2_ isotopic exchange. The tube is partly inserted into an oven as shown in the figure, with the Pt in the heated zone. We assume that the isotopic exchange takes place only at the bottom on the surface of the Pt catalyst, and the timescale of isotope exchange is much slower in the cold area in the absence of Pt, because there is not enough energy to break the H‐H bonds. Thus, the H_2_ will not re‐equilibrate significantly at the lower temperature during transfer to the bellow.

The procedure of the heating experiments was as follows. The heating tube was first evacuated and then filled with H_2_ to a certain pressure (between 400 and 1000 mbar absolute), taking into account the thermal expansion at the planned heating temperature. For most of the experiments, the transfer of H_2_ into the tube was done via a liquid nitrogen (LN_2_) trap to remove any traces of water and other potential impurities. The tube, already connected to the MAT 253‐Ultra instrument, was then inserted into the preheated oven, and, after the desired heating time, the gas was expanded into the sample bellow for analysis.

A number of heating experiments were performed at temperatures between 100 and 850°C. The heating duration varied between 15 min and 144 h; the short heating experiments were performed in order to determine the minimum time needed for isotopic equilibration at lower temperatures, and we assume that the equilibration time at higher temperatures is shorter. We used several gases with very different initial DD content, including the very enriched NAT‐360 gas with an initial ΔDD value of about +26000‰, with the expectation that after equilibration the temperature‐dependent ΔDD value should not depend on the initial isotopic composition, but only on the equilibration temperature.

### 2.7 Theoretical calculation of thermodynamic DD equilibration

The thermodynamic equilibrium constant for the three hydrogen isotopologues was calculated from molecular masses and internal partition sums,^40^ using the results of recent *ab initio* calculations of the energy levels of H_2_, D_2_ 41 and HD^42^ (Figure 6). The uncertainty of this calculation is estimated at about 1‰ or smaller for the entire temperature range between 5 and 1000 K. Overall, our results agree well with previous experimental and theoretical results;^4^, ^5^, ^28^, ^43^, ^44^, ^45^, ^46^ the only possible difference is with Gould et al^28^ at −190°C. This lets us safely conclude that the equilibrium constant is well known at the uncertainty level of 1‰ all over the temperature range between 100 and 2000 K.

### 2.8 Calculations and error estimation

δD and δDD values were calculated from the raw cps values using the definitions from section 2.1, considering the gas in bellow A as “reference”. For ΔDD calculation in the present work, conversion to the VSMOW scale was not necessary.

The analysis of one sample consists of a series of independent measurement cycles (usually between 10 and 20 cycles, see section 2.4) each giving one data point. Each such measurement series undergoes a manual quality check and the “bad” measurements are flagged (these are mostly due to errors in the automatic pressure adjust, or drift in the peak position). The resulting δD or δDD value for one sample is the average of the remaining “good” data points, and the standard error over these gives the measurement precision for that sample.

The ΔDD of a gas can in principle be estimated from the abundances of DD, HD and HH, using Equation 4 from section 2.1. However, application of Equation 4 requires knowledge of the absolute abundances of the three isotopologues but, as mentioned above, a calibrated standard for DD is not available. Therefore, we used as a reference for the ΔDD calculation one of the gases that were heated to 850°C. We chose (somewhat arbitrarily) the gas analyzed during Experiment 576, which had been heated to 850°C for 8 h, and we assigned it a ΔDD value of 19‰, which is the theoretically calculated value corresponding to this temperature. The sample ΔDD value was then calculated using the formula:

(5) $${\mathrm{∆DD}}_{\mathrm{sam}}=\frac{\left({\mathrm{\delta DD}}_{\mathrm{sam}-\mathrm{ref}}+1\right)}{{\left({\mathrm{\delta D}}_{\mathrm{sam}-\mathrm{ref}}+1\right)}^{2}}\times \left({\mathrm{∆DD}}_{\mathrm{ref}}+1\right)-1$$

The standard error of ΔDD was calculated by error propagation, from the errors of the δD and δDD values, considering the individual errors to be not correlated.

## 3 RESULTS

### 3.1 Precision of one sample measurement

**Figure 4:**
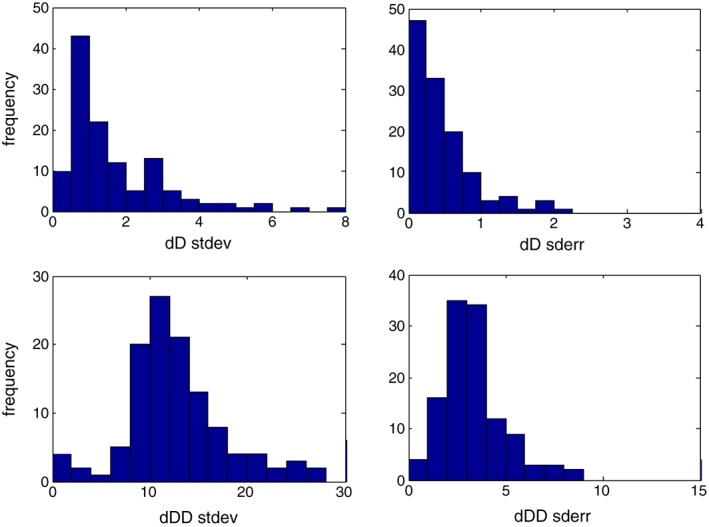
Histograms of the standard deviation and standard errors of the δD and δDD values for all experiments [Color figure can be viewed at wileyonlinelibrary.com]

Figure 4 shows the statistical distribution of the standard deviations and standard errors of all experiments, for both δD and δDD values. The standard deviation, which mainly represents the random error, is typically around 1‰ for δD and 15‰ for δDD values. The typical standard error of a complete measurement is better than 1‰ for δD and typically 2–6‰ for δDD values. These values are close to the statistical precision limits for δDD but not for δD values, for which the shot noise limit would be much lower because of the higher signal. This indicates that isotopic variability due to variations in instrument conditions associated with the “peak hopping” procedure is of a magnitude that lies between the counting statistics error for the δDD (standard deviation ~8‰) and δD (standard deviation ~0.1‰) values.

In general, instrument stability is one of the main factors limiting the measurement precision, with the peak position often drifting during the measurement. In addition, the decrease in pressure during a measurement cycle between two pressure adjust steps, which gradually becomes larger, starts to affect the results significantly at high bellow compression. Because of these effects, the precision of the average result does not increase any more after a relatively short number of measurements (compared with typical dual‐inlet measurements), as shown in the Allan variance plots in Figure S2 (Supplement 3, supporting information).

We tested measuring at lower source pressure, in order to assess the capability of measuring smaller samples. We observed as expected a small decrease in precision with pressure, but no systematic differences in the ΔDD results – see Figure S2 (Supplement 3, supporting information). A normal measurement (source pressure of 2.5e‐7 mbar) requires about 5 mL of H_2_, which is a pressure of 125 mbar in the 40‐mL bellow (all gas volumes are at standard laboratory conditions unless specified differently). We obtained good measurements even with a sample size of about 1.5 mL H_2_. In these measurements the source pressure was about half the normal pressure and measurements were started with the bellows partly compressed.

### 3.2 Medium‐term reproducibility

**Figure 5:**
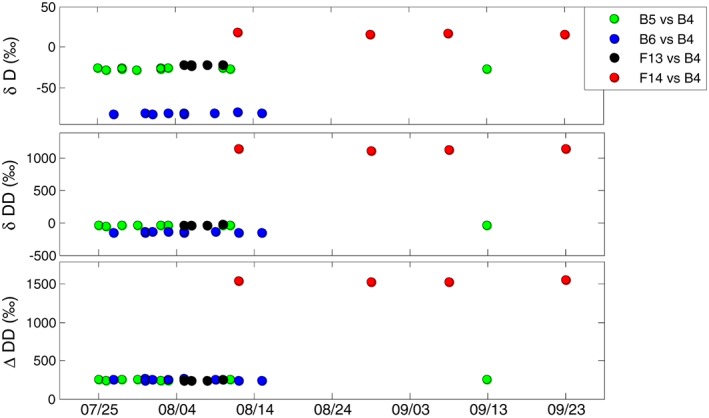
Stability of δD, δDD and ΔDD results of repeated measurements of several gases, over approx. 2 months. The error bars are smaller than the markers [Color figure can be viewed at wileyonlinelibrary.com]

Four samples were analyzed repeatedly over several weeks to months, sometimes under slightly different conditions, e.g. at different source pressure. The values of the δD, δDD versus the reference gas (B4), and the ΔDD values of these measurements are shown in Figure 5. The long‐term variations in the δD and δDD values (Table 2) are consistent with the within‐measurement precision, which means that there are no major additional errors affecting the measurement reproducibility in the longer term. The ΔDD values show a variability level similar to that of the δDD values, as expected, because the error in ΔDD is mainly determined by the error in the δDD value.

**Table 2:** Mean and standard deviations of δD, δDD and ΔDD values of long‐term repeated measurements for selected samples. The typical precision (the median of the measurement standard error, section 3.1) is shown for comparison

Sample	δD vs B4 mean	δD stdev	δDD vs B4 mean	δDD stdev	ΔDD mean	ΔDD stdev	n
B5	−26.5	0.9	−39.7	3.3	248.5	3.5	11
B6	−81.8	0.8	−142.9	4.7	252.5	7.4	12
F13	−21.8	0.5	−34.8	4.4	242.7	5.4	6
F14	+16.4	1.3	+1132.6	12.1	1543.2	12.4	4
Typical precision		0.4		3.9		2–6	

### 3.3 Sample inter‐compatibility

Several gases were analyzed using two different gases (B4 and B6) as “reference”. The relative δD and δDD values were then calculated for the pairs of gases, using each of the different references. The results are shown in Table 3; the italic values show the direct measurements versus the respective reference, while the other values are calculated from two measurements (e.g. the values for B5 vs B4 with B6 as reference were calculated from B5 vs B6 and B4 vs B6). The relative delta values of the pairs of samples are consistent when using the two references with different isotopic composition, which means that they are not reference dependent but represent the true property of the gases analyzed.

**Table 3:** Comparison of direct and indirect measurements of δD and δDD values for several pairs of gases. The italic values show the direct measurements

	B6 as ref (bellow A)		B4 as ref (bellow A)	
	δD	δDD	δD	δDD
B6 vs B4	*−82.0*	*−145.7*	*−81.8*	*−142.9*
B6 vs B5	*−56.9*	*−107.1*	−56.9	−107.7
B5 vs B4	−26.7	−43.3	*−26.5*	*−39.5*
F14 vs B4	17.3	1147.3	*18.2*	*1142.1*
F14 vs B5	45.1	1244.4	45.9	1230.2
F14 vs B6	*107.3*	*1503.1*	109.0	1499.3

### 3.4 Heating experiments

An overview of the heating experiments is given in Table 4. Some experiments are considered questionable (e.g. due to probable incomplete tubing flushing after analyzing samples very enriched in DD) and are flagged out. The rest of the experiments are considered further in this section.

**Table 4:** Overview of the heating experiments. For calculation of ΔDD, the gas from Exp. 576 (marked yellow) was used as a reference, with an assigned ΔDD value of +19‰. The experiments are grouped by gas and then in chronological order. The flags have the following meaning: 0 = good; 1 = questionable; 3 = room temperature, not equilibrated; 4 = short heating. Only data with flag = 0 were used for Figure 6, these are shown in italic [Color table can be viewed at wileyonlinelibrary.com]

Experiments						Results vs IMAU‐H2 bottle 4									Δ*DD calculation vs ref*			
Date	Exp.	Bottle	Gas	Temp (C)	Time (min)	dD mean	dD std	dD n	dD sderr	dDD mean	dDD std	dDD n	dDD sderr	Flag	dD (‰)	dDD (‰)	Δ*DD (‰)*	Δ*DD sderr*
16‐Aug‐17	521	Tube‐1‐1	NAT‐360	850	30	397.3	1.9	20	0.4	690.6	23.5	20	5.3	1	360.4	907.6	*50.3*	*2.9*
17‐Aug‐17	522	Tube‐1‐2	NAT‐360	850	30	293.9	1.2	10	0.4	488.4	23.3	10	7.4	3	259.7	679.4	*78.3*	*4.7*
*17‐Aug‐17*	*523*	*Tube‐2‐1*	*NAT‐360*	*850*	*280*	*383.3*	*1.9*	*10*	*0.6*	*622.4*	*20.8*	*12*	*6.0*	*0*	*346.8*	*830.7*	*28.5*	*3.4*
*17‐Aug‐17*	*524*	*Tube‐2‐2*	*NAT‐360*	*850*	*565*	*361.1*	*1.2*	*18*	*0.3*	*570.5*	*31.4*	*18*	*7.4*	*0*	*325.2*	*772.1*	*28.3*	*4.2*
*18‐Aug‐17*	*527*	*Tube‐4*	*NAT‐360*	*350*	*385*	*378.2*	*0.5*	*5*	*0.2*	*691.8*	*26.8*	*6*	*10.9*	*0*	*341.9*	*909.0*	*80.3*	*6.1*
*18‐Aug‐17*	*528*	*Tube‐4*	*NAT‐360*	*350*	*385*	*372.0*	*5.5*	*24*	*1.1*	*676.7*	*27.4*	*24*	*5.6*	*0*	*335.7*	*891.9*	*80.5*	*3.6*
29‐Aug‐17	561	Tube‐5‐1	NAT‐360	25	0	359.0	1.1	2	0.8	39741.9	341.8	2	241.7	3	323.1	44970.6	*25757.5*	*141.4*
30‐Aug‐17	562	Tube‐5‐1	NAT‐360	25	0	370.8	3.2	22	0.7	41244.5	463.0	28	87.5	3	334.6	46666.1	*26270.9*	*56.2*
30‐Aug‐17	563	Tube‐5‐2	NAT‐360	850	465	400.9	3.1	18	0.7	723.1	22.6	18	5.3	1	363.9	944.2	*65.0*	*3.1*
31‐Aug‐17	565	Tube‐5‐3	NAT‐360	850	1540	379.8	7.3	10	2.3	671.2	34.0	10	10.8	1	343.4	885.7	*64.8*	*7.0*
01‐Sep‐17	566	Tube‐5‐4	NAT‐360	850	2880	371.2	1.5	26	0.3	639.9	20.5	30	3.7	1	335.0	850.4	*57.9*	*2.1*
*05‐Sep‐17*	*567*	*Tube‐5‐5*	*NAT‐360*	*850*	*8640*	*353.1*	*1.0*	*22*	*0.2*	*529.3*	*29.8*	*28*	*5.6*	*0*	*317.4*	*725.5*	*13.2*	*3.3*
06‐Sep‐17	567	Tube‐6‐1	NAT‐360	850	15	381.2	4.4	29	0.8	597.0	13.1	8	4.6	4	344.7	801.9	*15.4*	*2.8*
*07‐Sep‐17*	*569*	*Tube‐6‐2*	*NAT‐360*	*850*	*1440*	*357.1*	*3.0*	*8*	*1.1*	*518.4*	*30.7*	*14*	*8.2*	*0*	*321.2*	*713.3*	*0.1*	*5.0*
*13‐Sep‐17*	*575*	*Tube‐7‐1*	*IMAU‐H2*	*25*	*15*	*10.1*	*1.6*	*12*	*0.5*	*31.6*	*11.4*	*12*	*3.3*	*0*	*−16.6*	*163.9*	*226.3*	*3.6*
*13‐Sep‐17*	*576*	*Tube‐7‐2*	*IMAU‐H2*	*850*	*480*	*27.1*	*2.0*	*13*	*0.6*	*−113.7*	*9.2*	*14*	*2.5*	*0*	*0.0*	*0.0*	*19.0*	*2.7*
*14‐Sep‐17*	*577*	*Tube‐8‐1*	*IMAU‐H2*	*850*	*10*	*27.0*	*3.5*	*14*	*0.9*	*−113.7*	*15.5*	*14*	*4.1*	*0*	*−0.1*	*0.0*	*19.3*	*4.5*
*14‐Sep‐17*	*578*	*Tube‐8‐2*	*IMAU‐H2*	*850*	*300*	*13.9*	*4.2*	*7*	*1.6*	*−119.2*	*14.5*	*8*	*5.1*	*0*	*−12.9*	*−6.2*	*39.3*	*6.2*
14‐Sep‐17	579	Tube‐9‐1	IMAU‐H2	650	15	23.5	1.2	18	0.3	−112.3	12.3	19	2.8	4	−3.6	1.6	*28.0*	*2.9*
*15‐Sep‐17*	*580*	*Tube‐9‐2*	*IMAU‐H2*	*650*	*720*	*9.3*	*1.1*	*14*	*0.3*	*−126.8*	*18.4*	*14*	*4.9*	*0*	*−17.3*	*−14.7*	*39.7*	*5.1*
15‐Sep‐17	581	Tube‐10‐1	IMAU‐H2	450	10	24.2	0.5	19	0.1	−92.1	13.9	20	3.1	4	−2.8	24.4	*49.8*	*3.1*
*15‐Sep‐17*	*582*	*Tube‐10‐2*	*IMAU‐H2*	*450*	*360*	*10.8*	*5.8*	*20*	*1.3*	*−118.8*	*15.5*	*19*	*3.5*	*0*	*−15.9*	*−5.7*	*46.1*	*4.5*
*16‐Sep‐17*	*583*	*Tube‐11–1*	*IMAU‐H2*	*350*	*780*	*22.6*	*1.7*	*20*	*0.4*	*−74.9*	*12.6*	*20*	*2.8*	*0*	*−4.4*	*43.8*	*73.0*	*3.0*
16‐Sep‐17	584	Tube‐12–1	IMAU‐H2	220	15	22.0	3.2	20.0	0.7	−46.7	16.6	20.0	3.7	4	−5.0	75.7	*107.1*	*4.1*
*17‐Sep‐17*	*585*	*Tube‐12–2*	*IMAU‐H2*	*220*	*660*	*11.4*	*3.5*	*17.0*	*0.8*	*−76.3*	*16.5*	*18.0*	*3.9*	*0*	*−15.3*	*42.2*	*95.2*	*4.4*
17‐Sep‐17	586	Tube‐13‐1	IMAU‐H2	100	25	15.2	1.7	20.0	0.4	33.1	16.5	19.0	3.8	4	−11.6	165.6	*215.9*	*4.0*
*18‐Sep‐17*	*587*	*Tube‐13‐2*	*IMAU‐H2*	*100*	*720*	*9.5*	*6.8*	*19.0*	*1.6*	*−22.2*	*20.3*	*19.0*	*4.6*	*0*	*−17.2*	*103.3*	*163.9*	*6.0*
*18‐Sep‐17*	*588*	*Tube‐14‐1*	*IMAU‐H2*	*100*	*540*	*16.5*	*2.1*	*20.0*	*0.5*	*−8.1*	*16.5*	*20.0*	*3.7*	*0*	*−10.3*	*119.2*	*164.3*	*3.9*
*18‐Sep‐17*	*589*	*Tube‐14‐2*	*IMAU‐H2*	*100*	*1260*	*12.7*	*0.6*	*16.0*	*0.1*	*−14.9*	*13.9*	*16.0*	*3.5*	*0*	*−14.1*	*111.5*	*165.2*	*3.6*
*19‐Sep‐17*	*590*	*Tube‐14‐3*	*IMAU‐H2*	*100*	*1740*	*10.2*	*1.6*	*20.0*	*0.3*	*−27.2*	*16.8*	*20.0*	*3.8*	*0*	*−16.5*	*97.6*	*156.3*	*4.0*
*19‐Sep‐17*	*591*	*Tube‐15–1*	*IMAU‐H2*	*100*	*70*	*20.2*	*1.3*	*14.0*	*0.4*	*−2.9*	*12.9*	*14.0*	*3.4*	*0*	*−6.7*	*125.1*	*162.1*	*3.6*
*20‐Sep‐17*	*592*	*Tube‐15–2*	*IMAU‐H2*	*100*	*750*	*17.6*	*1.6*	*19.0*	*0.4*	*−11.0*	*10.3*	*19.0*	*2.4*	*0*	*−9.2*	*116.0*	*158.5*	*2.6*
*20‐Sep‐17*	*595*	*Tube‐16–1*	*IMAU‐H2*	*100*	*27*	*16.6*	*0.7*	*23.0*	*0.1*	*−5.1*	*10.1*	*23.0*	*2.1*	*0*	*−10.3*	*122.6*	*167.8*	*2.2*
*21‐Sep‐17*	*596*	*Tube‐16–2*	*IMAU‐H2*	*100*	*720*	*13.6*	*0.9*	*10.0*	*0.3*	*−16.3*	*12.7*	*10.0*	*4.0*	*0*	*−13.2*	*109.9*	*161.5*	*4.2*
21‐Sep‐17	597	Tube‐17–1	IMAU‐H2	100	1	11.0	0.5	8.0	0.2	29.4	13.5	8.0	4.8	4	−15.6	161.5	*221.5*	*4.9*
21‐Sep‐17	598	Tube‐17–2	IMAU‐H2	100	15	18.1	3.3	25.0	0.7	29.6	14.3	24.0	2.9	4	−8.8	161.8	*204.9*	*3.4*
*26‐Sep‐17*	*612*	*Tube‐25–1*	*IMAU‐H2*	*850*	*70*	*32.2*	*0.7*	*14*	*0.2*	*−102.9*	*8.1*	*13*	*2.2*	*0*	*5.0*	*12.3*	*21.4*	*2.2*
*27‐Sep‐17*	*615*	*Tube‐25–2*	*IMAU‐H2*	*850*	*1260*	*16.8*	*0.7*	*11*	*0.2*	*−135.5*	*12.2*	*14*	*3.3*	*0*	*−10.1*	*−24.5*	*14.3*	*3.4*
22‐Sep‐17	599	B14	Flask 14	25		15.5	1.1	14.0	0.3	1142.4	23.3	14.0	6.2	3	−11.3	1417.4	*1519.9*	*6.5*
*22‐Sep‐17*	*600*	*Tube 18–1*	*Flask 14*	*850*	*40*	*28.1*	*1.4*	*16*	*0.4*	*−114.8*	*13.4*	*16*	*3.4*	*0*	*1.0*	*−1.2*	*15.8*	*3.4*
*22‐Sep‐17*	*601*	*Tube 18–2*	*Flask 14*	*850*	*350*	*−0.7*	*0.9*	*13*	*0.3*	*−165.9*	*12.4*	*13*	*3.4*	*0*	*−27.1*	*−58.8*	*13.1*	*3.7*
*22‐Sep‐17*	*602*	*Tube‐19–1*	*Flask 14*	*450*	*210*	*32.9*	*9.7*	*14*	*2.6*	*−83.9*	*13.3*	*14*	*3.6*	*0*	*5.7*	*33.6*	*41.4*	*6.3*
*23‐Sep‐17*	*603*	*Tube‐20‐1*	*Flask 14*	*100*	*480*	*20.5*	*4.1*	*18*	*1.0*	*−12.1*	*16.7*	*18*	*3.9*	*0*	*−6.5*	*114.7*	*150.7*	*4.6*
*23‐Sep‐17*	*605*	*Tube‐21–1*	*AP‐552*	*25*		*−83.5*	*1.8*	*19.0*	*0.4*	*−139.4*	*12.8*	*19.0*	*2.9*	*0*	*−107.7*	*−29.0*	*242.7*	*3.8*
*24‐Sep‐17*	*606*	*Tube‐21–2*	*AP‐552*	*100*	*810*	*−76.7*	*0.9*	*9*	*0.3*	*−173.4*	*10.2*	*10*	*3.2*	*0*	*−101.1*	*−67.3*	*176.3*	*4.1*
*24‐Sep‐17*	*608*	*Tube‐22–1*	*AP‐552*	*450*	*180*	*−64.5*	*0.9*	*20*	*0.2*	*−224.7*	*10.3*	*20*	*2.3*	*0*	*−89.2*	*−125.2*	*74.6*	*2.8*
*25‐Sep‐17*	*609*	*Tube‐22–2*	*AP‐552*	*250*	*600*	*−96.8*	*2.7*	*14*	*0.7*	*−256.3*	*14.3*	*14*	*3.8*	*0*	*−120.6*	*−160.8*	*105.7*	*5.2*
*25‐Sep‐17*	*610*	*Tube‐23‐1*	*AP‐552*	*850*	*60*	*−59.8*	*1.6*	*19*	*0.4*	*−237.3*	*15.5*	*19*	*3.6*	*0*	*−84.6*	*−139.4*	*46.5*	*4.3*
*26‐Sep‐17*	*611*	*Tube‐23‐2*	*AP‐552*	*850*	*1320*	*−79.1*	*8.4*	*12*	*2.4*	*−273.4*	*15.0*	*12*	*4.3*	*0*	*−103.4*	*−180.1*	*39.2*	*7.7*

Several experiments involved very short heating times (less than 30 min), in order to test the time needed for complete equilibration time at different temperatures. We observed incomplete equilibration only at 100°C, for heating times shorter than 30 min. At higher temperature the equilibration was faster, as shown by ΔDD not changing with continued heating. We consider therefore that a gas heated for longer than 30 min at a certain temperature is thermodynamically equilibrated at that temperature.

**Figure 6:**
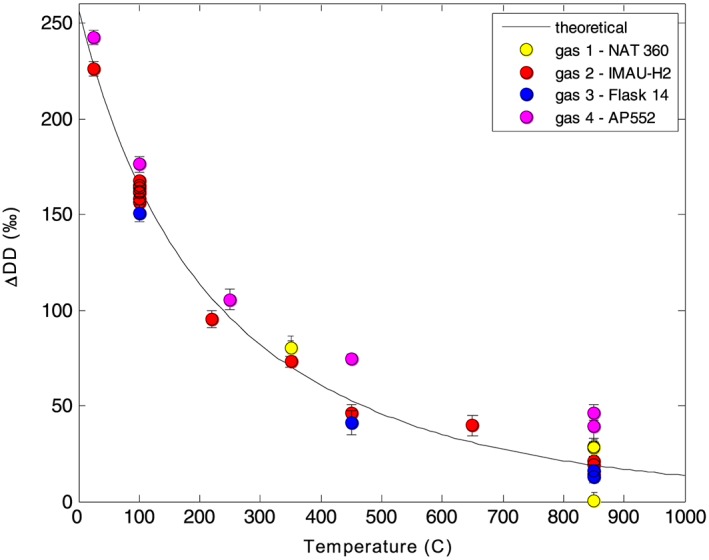
Results of heating experiments: Dependence of ΔDD on the equilibration temperature, compared with the theoretical results (solid line). The different H_2_ gases are shown in different colors. Gas 1 (NAT 360) is the one that before equilibration is very enriched in DD (+26000‰) [Color figure can be viewed at wileyonlinelibrary.com]

The results of the temperature equilibration experiments are shown in Figure 6, with different colors for different gases. Only heating times longer than 30 min are considered here. As already mentioned, we used four gases with different isotopic composition; one of these has an extreme enrichment in DD, with an initial ΔDD value of about +26000‰. We included this gas in order to verify that starting with a very anomalous composition does not influence the final ΔDD value after equilibration, and the results prove that this is the case. Indeed, the results of all four gases are similar. Gas 4 seems to have generally higher ΔDD values – the cause is presently unknown, but this was the last tested gas, during a period when a contamination with N_2_ was also observed, indicating possible air leakage into the sample.

The theoretical temperature dependence curve, calculated as described in section 2.7, is also shown in Figure 6. The experimental results fall well around this line, except for Gas 4 as mentioned above. The scatter around the calculated temperature curve has been estimated as the standard deviation of the residuals. The observed scatter is between 4 and 12‰ for the different gases, which is comparable with the scatter of repeated measurements as shown in Table 2.

As explained above, one of the experimental results where H_2_ was heated to 850°C was used as reference for the calculation of the ΔDD value. The results would be slightly different if we chose the gas from another experiment as a reference; however, changing the reference would mainly introduce a “vertical” shift of all values in Figure 6, but would not change the shape of the curve. Therefore, we can confidently state that the experimental evolution of ΔDD with temperature is as expected.

The fact that the experimental results reproduce the theoretical dependence of ΔDD on equilibration temperature: (1) provides a convincing validation of our measurement method; (2) demonstrates that no significant re‐equilibration occurs inside the instrument; and (3) implies that this method holds potential for producing a calibration scale.

### 3.5 Mass stability

The peak position stability is essential for the H_2_ measurements, because the flat shoulder part of the HD and DD peaks next to the interfering adduct peaks is very narrow (Figure 1). Moreover, the peak position has to be precisely reproduced after each peak hopping, i.e. for each individual determination of an ion signal. At the low field strengths required for analysis of the light isotopologues of H_2_, the MAT 253‐Ultra is in principle able to find the narrow plateaus (peak shoulders) after each peak jump. However, at the sub‐mu level needed for the measurements presented here, the peak stability was somewhat unpredictable, with stable time periods followed by unstable ones or even large jumps that we could not explain. Figure S1 (Supplement 3, supporting information) shows the peak position registered at the beginning of each experiment, over the several months of experiments presented here.

### 3.6 Storage in bellows and containers

The stability of the sample (see section 3.2) shows that the containers that we used are stable for H_2_ traditional and clumped isotope measurements over the time range of a few months, and thus suitable for storage of pure H_2_ samples.

In several tests we stored H_2_ in bellows overnight, and we measured δD and δDD values before and after. We also tested the storage volumes between bellows and the valve to the source, storing the H_2_ for about 1 day and measuring before and after (tests 499 and 500). No significant difference was observed in any of these, which suggests that no re‐equilibration takes place inside the instrument over time intervals of several hours.

## 4 DISCUSSION, CONCLUSIONS AND OUTLOOK

We established the first measurement method of ΔDD in H_2_ using the MAT 253‐Ultra instrument. A high‐precision isotope measurement at the counting statistics level for ΔDD is realized by sequential recording of the three isotope masses for HH, HD and DD with individual, different collectors.

### 4.1 Precision

The ΔDD precision of 2–6‰ obtained for a sample of 5 mL is sufficient to detect the expected differences between environmental samples, and also temporal and spatial variations in atmospheric H_2_. The precision depends on the number of usable data points that can be obtained for one sample, which is limited by the peak position stability, and by the larger pressure decrease between two pressure‐adjust events at low bellow volume (high compression).

It is evident that instrument stability is the key requirement when attempting isotope ratio measurements sequentially and under dynamically changing intensities rather than simultaneously. The results demonstrate that the MAT 253‐Ultra instrument is generally sufficiently stable to allow measurements of a signal of merely 30 cps for DD over several hours and under dynamic conditions near the counting statistics level. The system is generally able to repeatedly position the ion beams into the collectors in a reproducible manner at a precision of 0.1 mu at the extremely low magnetic field strengths that are required for these light molecules.

### 4.2 Sample size

The H_2_ sample size needed for a measurement is decisive for the types of samples that will be possible to analyze. So far, we have demonstrated that we can achieve systematically good results with a sample size of 5 mL H_2_; although the precision decreases somewhat, acceptable results were obtained when the sample size was reduced to 1.5 mL H_2_. Considering atmospheric air with a H_2_ mole fraction of 500 ppb, an air volume of 10 m^3^ is needed to obtain a volume of H_2_ of 5 mL. Geologic and microbial gas samples can have up to percent levels of H_2_; thus, much smaller quantities of gas would be needed in such cases.

### 4.3 Reproducibility and stability

As shown in section 3.2, repeated measurements over several weeks to months of the same sample give stable results, consistent with the measurement precision. This fulfills an essential validity condition for our measurements. In addition, this shows that pure H_2_ samples can be stored for months in containers without significantly altering their ΔDD signature.

### 4.4 Instrumental improvements

Some of the current limitations in precision and sample size needed could be overcome with relatively simple changes in the instrument software. Implementing a procedure of peak position correction transfer in the software (i.e. scan a peak, define a correction, and apply it to other peaks), and/or a peak centering based on a shoulder scan, would allow the peak positions to be adjusted during a measurement, which would result in longer usable measurement sequences. A continuous pressure adjustment would improve/allow measurements at lower bellow sizes, and thus with lower sample size. With these improvements we estimate that the measurements on a sample size lower than 1 mL should be possible, with a precision of a few per mill. In the case of atmospheric samples, for a quantity of 1 mL H_2_, one needs 2 m^3^ air (2000 L) at the normal atmospheric H_2_ mole fraction of 500 ppb.

### 4.5 Calibration

Currently, there are no available standards with known δDD (or ΔDD) values in H_2_ at natural levels; however, such standards will be needed if we aim to analyze H_2_ in natural samples. We intend to use the heating method presented here for producing, in the near future, calibration gases with known ΔDD anomaly. The experiments so far prove that the method works in principle. To define a calibration scale, we need to improve the reproducibility of this method, and to validate it by independent methods, e.g. producing H_2_ by electrolysis from waters with different δD values, and/or mixing gases with different δD and δDD compositions.

### 4.6 Extraction of H_2_ from air (gas) samples and its potential utility

In order to analyze natural samples, it is necessary to extract pure H_2_ from a variety of gases or air. Extracting H_2_ is more difficult than for most other common gases, because H_2_ has a very low condensation point. The method foreseen includes a combination of cryogenic and gas chromatographic separation steps. The use of He as a carrier gas is not possible for this application because it is very difficult to separate from H_2_ afterwards. If significant amounts of He remain in the H_2_ sample, the DD peak would be located on the He peak tail.

One of our main interests is measuring ΔDD of atmospheric H_2_, but very interesting applications are related to other fields like geology and (micro)biology. For H_2_ formed in thermodynamic equilibrium, the ΔDD anomaly will be linked to the temperature of formation. We expect this to be the case for some geologic samples, for example, in gas emitted from H_2_‐rich seeps and volcanoes. On the other hand, the H_2_ of microbiological origin (e.g. fermentation or N_2_ fixation) is not necessarily at thermodynamic equilibrium; such a phenomenon has been observed for CH_4_.^47^, ^48^ In that case, it is likely that the ΔDD anomaly (when the formation temperature is known) will inform us about processes taking place at the molecular level during H_2_ formation.

## Supporting information

Supporting Info Item.

Supporting Info Item.

Figure S1. Peak position stability over time for HD and DD.
Figure S2. Allan deviation plots examples for several experiments. Each data series shows the evolution of the standard deviation of the mean, with the addition of new data points. In general, the error decreases over the first ~ 15 data points, after which it stabilizes or even increases again. This shows that increasing the number of measurements after this does not improve the precision of the final result significantly.
Figure S3. Dependence of the standard error of the mean sample result on the Pressure adjust setting, which is implemented as target intensity for the Mass 2 signal. The “normal” working source pressure is 2.5e‐7 mbar, and this corresponds to about 9e9 cps.
