# Supplementary material for: H2 clumped isotope measurements at natural isotopic abundances
Source: Rapid Commun Mass Spectrom. 2019 Jan 11;33(3):239–51. doi: 10.1002/rcm.8323 (PMC6590658; doi:10.1002/rcm.8323)
Supplement: Supplementary file 3 — Figure S1. Peak position stability over time for HD and DD. Figure S2. Allan deviation plots examples for several experiments. Each data series shows the evolution of the standard deviation of the mean, with the addition of new data points. In general, the error decreases over the first ~ 15 data points, after which it stabilizes or even increases again. This shows that increasing the number of measurements after this does not improve the precision of the final result significantly. Figure S3. Dependence of the standard error of the mean sample result on the Pressure adjust setting, which is implemented as target intensity for the Mass 2 signal. The “normal” working source pressure is 2.5e‐7 mbar, and this corresponds to about 9e9 cps. [file RCM-33-239-s003.docx]

Figure S1. Peak position stability over time for HD and DD.


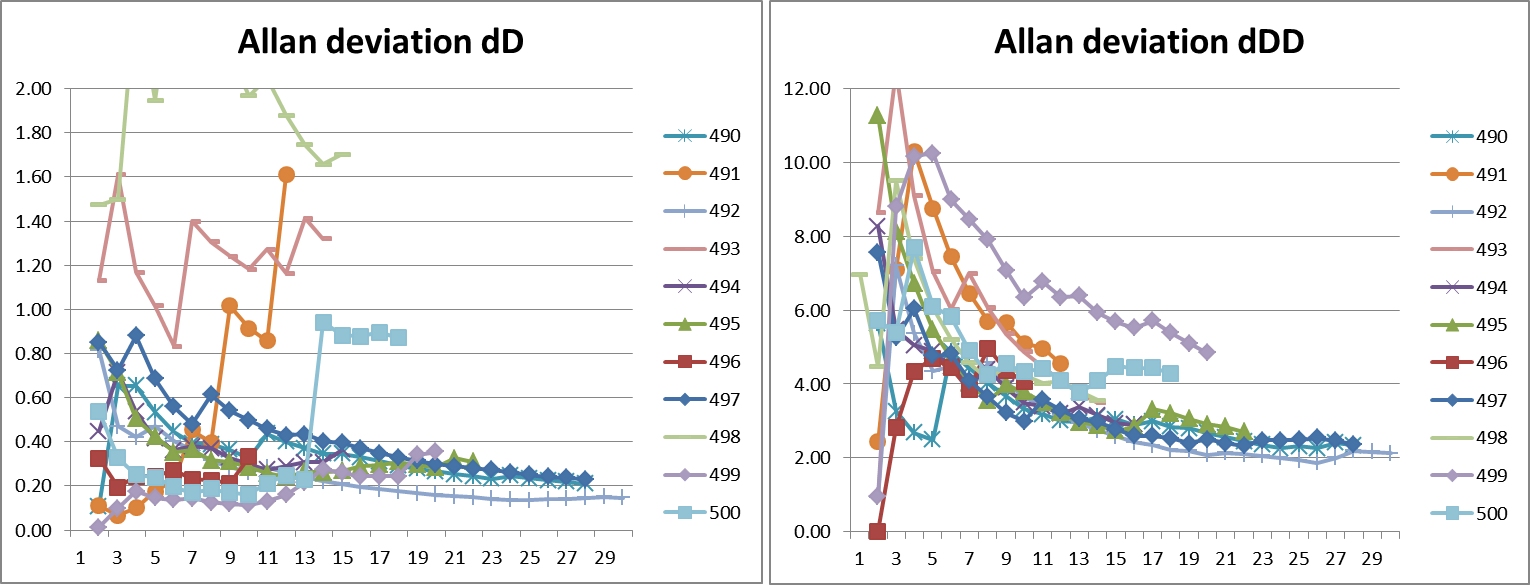


Figure S2. Allan deviation plots examples for several experiments. Each data series shows the evolution of the standard deviation of the mean, with the addition of new data points. In general, the error decreases over the first ~ 15 data points, after which it stabilizes or even increases again. This shows that increasing the number of measurements after this does not improve the precision of the final result significantly.


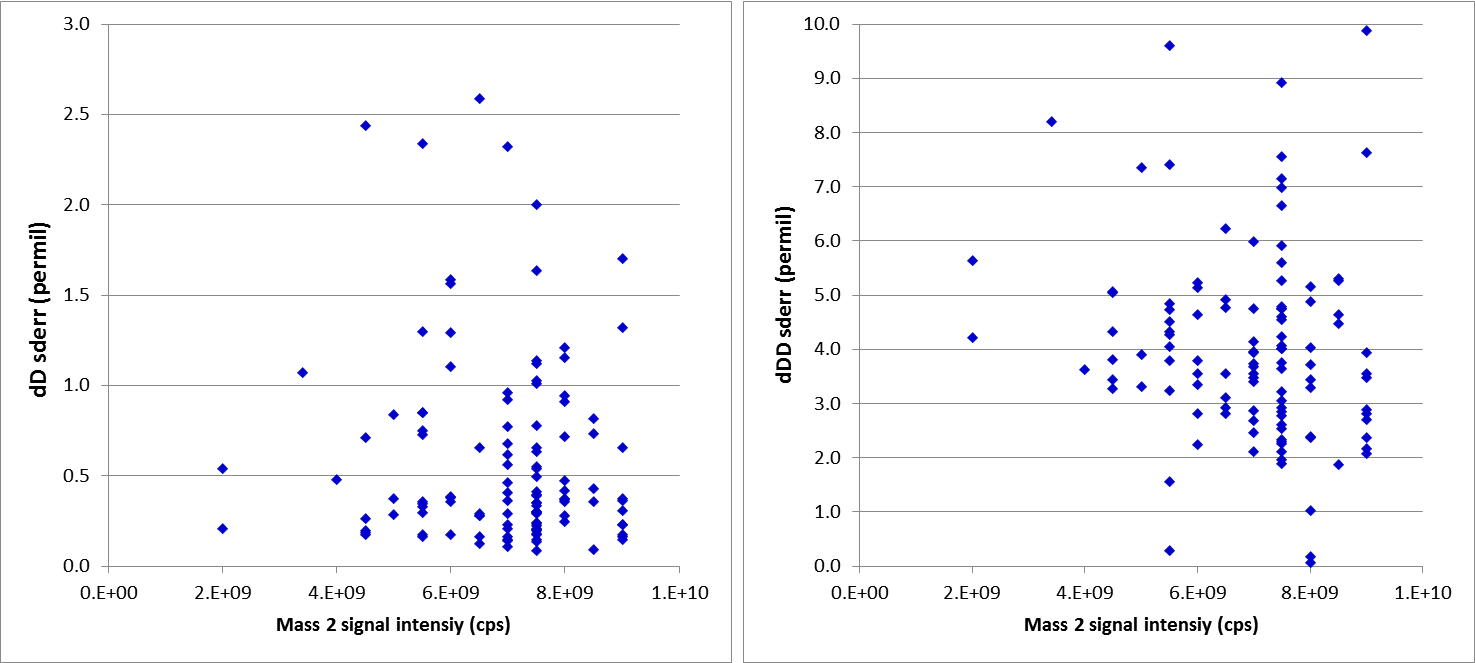


Figure S3. Dependence of the standard error of the mean sample result on the Pressure adjust setting, which is implemented as target intensity for the Mass 2 signal. The “normal” working source pressure is 2.5e-7 mbar, and this corresponds to about 9e9 cps.
